# Restored immune cell functions upon clearance of senescence in the irradiated splenic environment

**DOI:** 10.1111/acel.12971

**Published:** 2019-05-31

**Authors:** Lina Palacio, Marie‐Lyn Goyer, Damien Maggiorani, Andrea Espinosa, Norbert Villeneuve, Sara Bourbonnais, Gaël Moquin‐Beaudry, Oanh Le, Marco Demaria, Albert R. Davalos, Hélène Decaluwe, Christian Beauséjour

**Affiliations:** ^1^ Centre de recherche du CHU Ste‐Justine Montreal Quebec Canada; ^2^ Département de pharmacologie et physiologie, Faculté de Médecine Université de Montréal Montreal Quebec Canada; ^3^ European Research Institute for the Biology of Aging (ERIBA), University Medical Center Groningen (UMCG) University of Groningen Groningen The Netherlands; ^4^ Buck Institute For Research on Aging Novato California; ^5^ Département de Pédiatrie, Faculté de Médecine Université de Montréal Montreal Quebec Canada

**Keywords:** ionizing radiation, p16^INK4a^, senescence, senescent‐associated secretory phenotype, spleen, T cell

## Abstract

Some studies show eliminating senescent cells rejuvenate aged mice and attenuate deleterious effects of chemotherapy. Nevertheless, it remains unclear whether senescence affects immune cell function. We provide evidence that exposure of mice to ionizing radiation (IR) promotes the senescent‐associated secretory phenotype (SASP) and expression of p16^INK4a^ in splenic cell populations. We observe splenic T cells exhibit a reduced proliferative response when cultured with allogenic cells in vitro and following viral infection in vivo. Using p16‐3MR mice that allow elimination of p16^INK4a^‐positive cells with exposure to ganciclovir, we show that impaired T‐cell proliferation is partially reversed, mechanistically dependent on p16^INK4a^ expression and the SASP. Moreover, we found macrophages isolated from irradiated spleens to have a reduced phagocytosis activity in vitro, a defect also restored by the elimination of p16^INK4a^ expression. Our results provide molecular insight on how senescence‐inducing IR promotes loss of immune cell fitness, which suggest senolytic drugs may improve immune cell function in aged and patients undergoing cancer treatment.

## 1 INTRODUCTION

Cellular senescence is a complex phenotype observed in diverse tissues at distinct developmental stages (He & Sharpless, 2017). In adults, senescence likely acts to irreversibly prevent proliferation of damaged cells (Chen et al., 2005; Cosme‐Blanco et al., 2007; Feldser & Greider, 2007). Senescent cells appear during chronological aging, aberrant oncogene expression, and exposure to DNA damaging agents (Bartkova et al., 2006; Chen, Fischer, Reagan, Yan, & Ames, 1995). Without a universal marker, identification of senescent cells in tissues remains challenging. Expression of the tumor suppressor p16^INK4a^ increases with age in numerous mouse and human tissues and, thus, considered a reliable marker (Krishnamurthy et al., 2004). Exposure to ionizing radiation (IR) leads to delayed increase in p16^INK4a^ expression in mice tissues and cancer‐treated patients (Le et al., 2010; Marcoux et al., 2013; Sanoff et al., 2014).

Senescent cells accumulate in tissues and secrete a range of cytokines, chemokines, and proteases known as the senescence‐associated secretory phenotype (SASP) (Coppe et al., 2008; Kuilman et al., 2008). Why senescent cells accumulate in vivo remains unclear. One theory suggests senescence accumulates with a decline in immune functions with age. Several studies indicate senescent cell immune clearance in liver and tumorigenic tissues (Kang et al., 2011; Sagiv et al., 2013; Toso et al., 2014; Xue et al., 2007). While senescent cells support wound healing, accumulation of senescent cells also appears to contribute to tumor growth and development of age‐associated diseases (Coppe, Kauser, Campisi, & Beausejour, 2006; Krtolica, Parrinello, Lockett, Desprez, & Campisi, 2001; Oubaha et al., 2016). Significantly, genetic or pharmacological elimination of senescent cells reverses the onset of aging and associated pathologies in mice (Baker et al., 2016; Farr et al., 2017; Jeon et al., 2017; Ogrodnik et al., 2017; Schafer et al., 2017). Removing senescent cells reduces some side effects of chemotherapy and mitigate IR‐induced premature aging in murine hematopoietic stem cells (Chang et al., 2016; Demaria et al., 2017).

Substantial data demonstrate the impact of aging on the immune system promotes increased susceptibility to infection (Haynes & Swain, 2012; Kogut, Scholz, Cancro, & Cambier, 2012; Liu et al., 2011; Nikolich‐Zugich, 2014). Several groups demonstrate aged individuals exhibit attenuated immune adaptive response, particularly reduced proliferation of T cells and dendritic cell function (You, Dong, Mann, Knight, & Yaqoob, 2014). Deletion of p16^INK4a^ in T cells enhanced antigen‐specific immune response, which suggest senescence promotes an intrinsic defect in aged T cells (Liu et al., 2011). Moreover, mice lacking the expression of lamin A, a nuclear scaffolding protein, present an accelerated aging phenotype with immune deficiencies (Xin, Jiang, Kinder, Ertelt, & Way, 2015). In comparison, whether IR‐induced senescence has a long‐term impact on the immune system is less defined. A recent study showed mice irradiated (up to 4 Gy) at a young age failed to impair immune functions at old age (Pugh et al., 2016). Of note, the study compared irradiated aged mice (19 months) with age‐matched nonirradiated counterparts which already exhibit diminished immune function.

We previously observed irradiated mice developed impaired lymphopoiesis in the bone marrow, an effect both cellular nonautonomous and dependent on p16^INK4a^ (Carbonneau et al., 2012). Our current study sought to investigate whether IR‐induced p16^INK4a^ expression interfered with immune cell function. Using a previously described senescence mouse model (Demaria et al., 2014), we show that senescence‐inducing IR impairs immune cell function in the splenic environment, an effect partially driven by the SASP and reversible with clearance of p16^INK4a^‐positive senescent cells.

## 2 RESULTS

### 2.1 Exposure to IR induces features of senescence in the spleen

We previously showed that exposure to IR led to delayed (6–8 weeks) p16^INK4a^ expression in distinct mice tissues, including the spleen (Le et al., 2010; Palacio, Krishnan, Le, Sharpless, & Beausejour, 2017). The cause for expression delay remains unclear but it protects mice against cancer progression (Palacio et al., 2017). No data currently demonstrate whether IR‐induced p16^INK4a^ expression coincides with a senescence phenotype or/and whether it promotes adverse effects on splenic cell function. We sought to answer an important question. What effect does senescence‐induced IR have on immune and splenic function?

**Figure 1:**
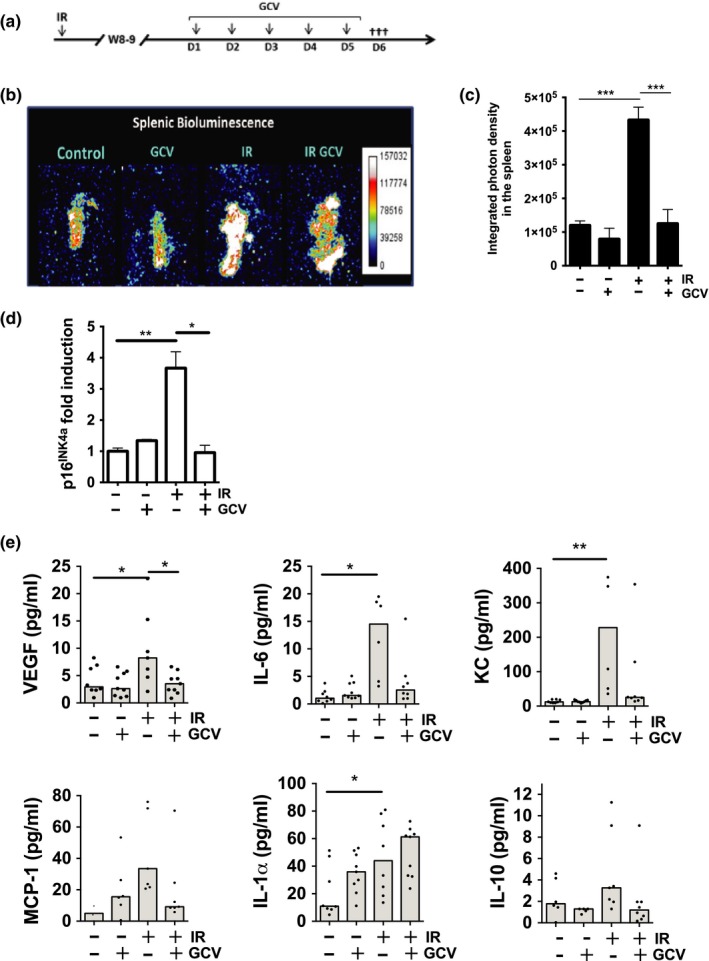
Exposure of mice to IR induces p16^INK4a^ expression and SASP in the spleen. (a) Schematic of the experimental design. Briefly, 12‐week‐old p16‐3MR mice were exposed to 6.5 Gy total body irradiation, and 8 to 9 weeks later, mice were treated or not with GCV for 5 consecutive days to eliminate *p16^INK4a^*‐positive cells. (b) One day after the last GCV treatment, mice were injected *i.p*. with coelenterazine (CTZ), and 14 min later, mice were sacrificed. Spleens were surgically removed to quantify the luminescence. Representative photographs are shown. (c) Shown is the average integrated photon density emitted from p16‐3MR mice exposed (+) or not (−) to IR and treated (+) or not (−) with GCV. (d) Quantification of endogenous *p16^INK4a^* mRNA levels as determined by qPCR from full spleen lysates. 18S ribosomal RNAs was used as an internal control. (e) Expression levels of VEGF, IL‐6, KC, MCP‐1, IL‐1α, and IL‐10 from splenocyte lysates as detected by multiplex array. Shown is the median analyzed by one‐way ANOVA ***p < 0.001; **p < 0.01; *p < 0.05; *n* = 5–8 mice per group

To answer these questions, we first exposed mice to total body irradiation at a sublethal dose of 6.5 Gy, the maximum tolerated dose for mice to survive without requiring a bone marrow transplant (Figure 1a). Due to the delay in p16^INK4^ expression, we waited 8 weeks post irradiation to allow increase in p16^INK4a^ expression and restrict the time required to regain steady spleen cellularity. We could not detect one marker of senescence (senescence‐associated β‐galactosidase) in irradiated spleen tissue sections. However, we observed an increase in p16^inK4a^ and SASP factors by qPCR along with a decrease in lamin B1 expression in macrophages (Figure S1A and B). Furthermore, we detected persistent DNA damage in stromal splenic cells, which did not appear in macrophages and hematopoietic cells (Figure S1C and D). Of note, splenic cell counts never completely reached levels observed prior to irradiation (Figure S2). We used p16‐3MR mice, in which p16^INK4a^‐positive cells are visualized and eliminated upon the administration of coelenterazine and ganciclovir (GCV), respectively (Demaria et al., 2014). Using luminescence reporter activity and qPCR data, we found p16^INK4a^ expression increased threefold to fourfold in irradiated spleens and significantly reduced in mice injected with GCV (Figure 1b–d). We collected splenic cell lysates to measure secretion of SASP markers (IL‐1a, IL‐6, MCP‐1, KC, and VEGF). The marker's concentration increased following irradiation and diminished in GCV‐treated mice, except for IL‐1a (Figure 1e). Although the SASP typically reflects inflammation, we observed a slight increase in IL‐10 secretion, a cytokine with pleiotropic immunosuppressive effects (Figure 1e).

### 2.2 Attrition of CD3^+^ and B220^+^ cell populations in the irradiated spleen

**Figure 2:**
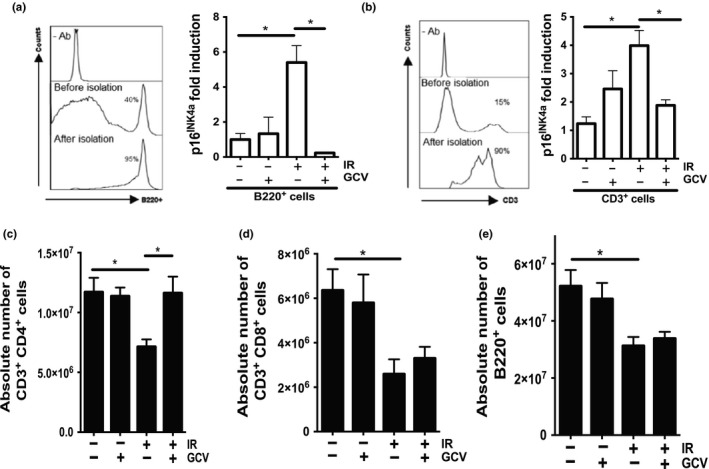
Attrition of T‐ and B‐cell populations in the irradiated spleen. (a, b) Shown are the purity (left panels) and quantification of *p16^INK4a^* mRNA levels (right panels) of isolated B220^+^ and CD3^+^ cell populations as determined by flow cytometry and qPCR, respectively. 18S ribosomal RNA was used as an internal control. (c–e) Quantification by flow cytometry of the absolute cell counts for CD3^+^CD4^+^, CD3^+^CD8^+^, and B220^+^ populations per full spleen collected from mice treated as indicated. Cell counts were determined 1 day following the last injection of GCV. Shown is the average ± *SEM*. The *ρ* value was determined by a one‐way ANOVA. *p < 0.05. *n* = 5–7 mice per group

A few days following IR, we detected substantial cell death in splenic T and B cells which also occurred in other cell populations to a lesser extent (Figures S2 and S3). A result consistent with the fact that lymphocytes constitute a highly radiosensitive cell population. We asked whether IR induced p16^INK4a^ in lymphocytes and whether this impacted their absolute number after reconstitution. We collected spleens from control, irradiated mice untreated/treated with GCV and dissociated at the single‐cell level. We isolated cell populations with magnetic columns (~90% purity as determined by flow cytometry) and extracted mRNA for qPCR analysis. We found p16^INK4a^ gene expression elevated fourfold in CD3^+^ cells and fivefold in B220^+^ cells (Figure 2a,b). These levels are similar to data from aged spleen‐derived lymphocytes (Krishnamurthy et al., 2004). Absolute cell counts diminished approximately 50% 8 weeks post‐IR for both CD3^+^ (CD4^+^ and CD8^+^) and B220^+^ cell populations (Figure 2c–e). While GCV injections effectively eliminated p16^INK4a^ expressing cells in both populations, only the CD3^+^CD4^+^ fraction exhibited cell levels observed in nonirradiated mice.

### 2.3 Impaired proliferation of T cells depends on the irradiated splenic environment

**Figure 3:**
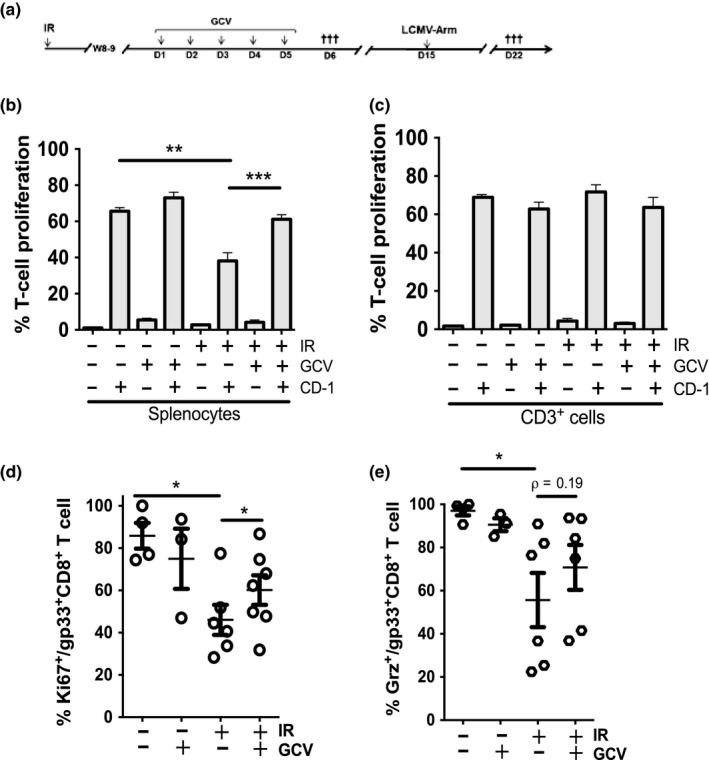
IR impairs T‐cell proliferation in vitro and in vivo. (a) Schematic of the experimental design. 12‐week‐old p16‐3MR mice were exposed to 6.5 Gy total body irradiation, and 8–9 weeks later, mice were treated or not with GCV for 5 consecutive days. On day 6 (D6), mice were sacrificed and splenocytes were collected and labeled with CFSE. Alternatively, on day 15 (D15), mice were injected with the LCMV‐Arm and sacrificed 1 week later. (b, c) The proportion of CD3^+^ cell undergoing proliferation following an allogenic stimulus was determined by flow cytometry. In panel b, the proliferation of T cells was determined from gated CFSE‐CD3^+^ cells from p16‐3MR splenocyte responder cells mixed with CD‐1 stimulator splenocytes (ratio 1:2). In panel c, the proliferation of T cells was determined from gated CFSE‐CD3^+^ from purified CD3^+^ responder cells mixed with CD‐1 stimulator splenocytes (ratio 1:2). (d) Quantification of the capability of splenic T cells to proliferate in vivo following the injection of mice with the LCMV‐Arm. Shown is the proportion of gp33^+^CD8^+^ T cells undergoing LCMV‐specific proliferation as determined by flow cytometry for the expression of the Ki67 proliferation marker. (e) Shown is the proportion of gp33^+^CD8^+^ T cells expressing granzyme B (Grz) as determined by flow cytometry. For all graphs, the average ± *SEM* is shown from *n* = 5–7 mice except for panels d and e where each dot represents counts from an individual mouse. The *ρ* value was determined by a one‐way ANOVA, ***p < 0.001; **p < 0.01; *p < 0.05

We next explored the impact of p16^INK4a^ expression on the proliferative potential of irradiated splenic T cells. To address this question, we conducted a mixed lymphocyte reaction (MLR) with splenocytes from untreated or treated p16‐3MR mice (Figure 3a, left segment) and freshly irradiated allogenic splenocytes obtained from a CD‐1 mouse. A one‐way MLR relies on the ability of “responder” cell (i.e., T cells within p16‐3MR splenocytes) activation by allogenic HLA molecules displayed by “stimulator” cells (i.e., antigen‐presenting cells in allogenic CD‐1 splenocytes). The one‐way MLR allowed us to restrict irradiated allogenic splenocytes to act as stimulators but not responders. Splenic T cells from p16‐3MR mice irradiated 8–9 weeks earlier exhibit approximately 40% reduced proliferation compared to splenic T cells from nonirradiated mice (~65%–70% vs. ~35%–40%, see Figure 3b). Significantly, the proliferation of splenic T cells was restored in irradiated mice treated with GVC (Figure 3b). In contrast, magnetically purified T cells (not full splenocytes) did not demonstrate improved proliferative capacity (Figure 3c).

To confirm this result, we measured the capacity of splenic T cells to proliferate in vivo in response to an acute lymphocytic choriomeningitis virus Armstrong (LCMV‐Arm) infection (Figure 3a, right segment). Seven days post viral infection, we detected reduced proliferation in a subset (CD8^+^gp33^+^, a dominant epitope of LCMV‐Arm) as measured by Ki67 expression (Figure 3d). GCV injection prior to viral infection promoted increased proliferation of antigen‐specific T cells. However, GCV did not significantly impact (*p* = 0.19) CD8^+^gp33^+^ T cells to secrete granzyme B in response to the LCMV‐Arm infection (Figure 3e). At the time of sacrifice, all mice had eliminated the virus showing no difference in serum (data not shown).

**Figure 4:**
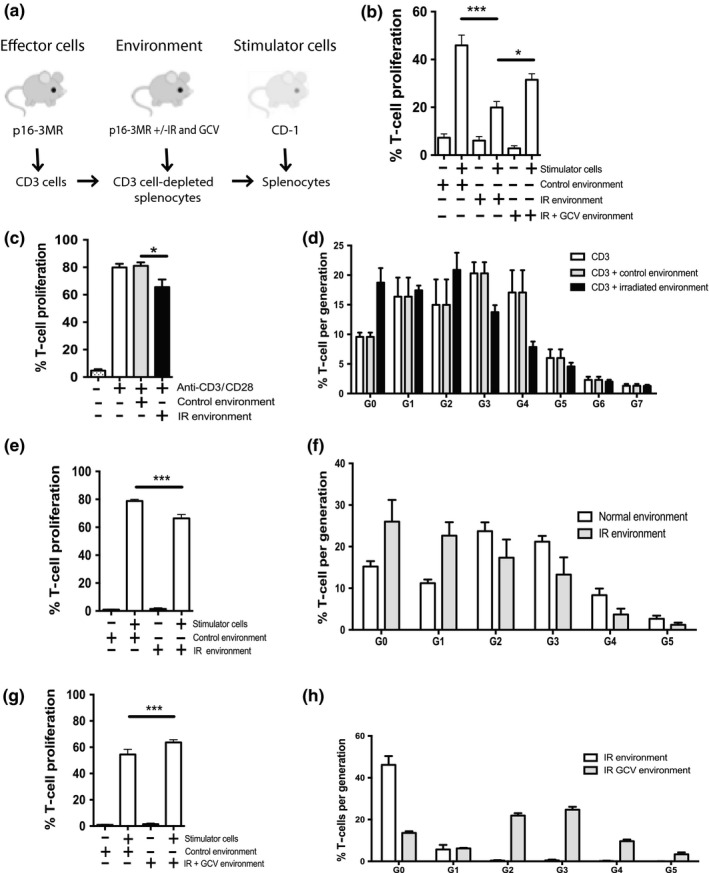
The irradiated splenic environment impairs T cell proliferation. (a) Schematic of the experimental design. CD3^+^ effector cells were isolated by negative selection from the spleens of p16‐3MR mice and labeled with CFSE. The splenic environment corresponds to CD3^+^ T cell‐depleted splenocytes from p16‐3MR mice previously (8–9 weeks) exposed (+) or not (−) to IR and treated or not with GCV. Stimulator cells were freshly irradiated (30 Gy) allogenic splenocytes collected from a CD‐1 mouse. (b) Shown is the proportion of effector CD3^+^ cell undergoing proliferation in the presence of the indicated splenic environment following an allogenic stimulus (CD‐1 stimulator). T cell proliferation was determined by flow cytometry (CFSE dilution). (c) Shown is the proportion of effector CD3^+^ cell undergoing proliferation following stimulation with anti‐CD3/anti‐CD28‐coated beads in the lower well of a Transwell plate with the indicated (control or IR) splenic environment in the top well. (d) Quantification of the number of cell generation as determined by flow cytometry by gating for each CFSE dilution peak obtained from the proliferation conditions shown in panel C. (e) Shown is the proportion of CD3^+^ cell undergoing proliferation following an allogenic stimulus (CD‐1 stimulator) in the lower well of a Transwell plate with the indicated splenic environment (control or IR) in the top well. (f) Quantification of the number of cell generation as determined from the proliferation conditions shown in panel E. (g) Shown is the proportion of CD3^+^ cell undergoing proliferation following an allogenic stimulus (CD‐1 stimulator) in the lower well of a Transwell plate and the indicated splenic environment (IR or IR + GCV) in the top well. (h) Quantification of the number of cell generation as determined from the proliferation conditions shown in panel g. Shown is the average ± *SEM* from *n* = 5–7 mice. Data were analyzed by one‐way analysis of variance (ANOVA). ***p < 0.001; *p < 0.05

Overall, these results demonstrate that IR does not impair intrinsic proliferation of splenic T cells. Irradiated splenocytes appear to compromise T‐cell proliferation in vitro and in vivo which suggests the splenic environment, either cells or their secretome, interferes with T‐cell proliferation. To test this notion, we performed a MLR with CD3^+^ effector T cells purified from p16‐3MR mice and stimulator cells from freshly irradiated CD‐1 splenocytes. We placed effector and stimulator cells in the presence of an “environment” of CD3^+^‐depleted splenocytes isolated from p16‐3MR mice (Figure 4a). This experimental setup confirmed that the presence of a previously (8–9 weeks) irradiated splenic environment compromised the proliferative capacity of purified T cells (Figure 4b). Additionally, we partially restored T cell proliferation when we used splenic environment from GCV‐treated mice with GCV (Figure 4b).

To identify whether irradiated splenocytes or their secretome proved detrimental to T cell proliferation, we performed a modified MLR whereby we separated effector/stimulator cells from splenocytes using a transwell. Thus, preventing cellular interaction allowed us to determine whether the splenic environment (secretome) effectively interfered with proliferation. Using beads coupled to anti‐CD3 and anti‐CD28 antibodies as stimulator, we found that delay in T cell proliferation only required exposure to irradiated splenocyte secretome (Figure 4c,d). We confirmed the negative impact of irradiated secretome on T cell proliferation, testing allogenic splenocytes collected from a CD1 mouse as stimulator. We similarly found proliferation of T cells diminished in the presence of an irradiated splenic environment (Figure 4e,f) and increased with GCV treatment (Figure 4g,h). Of note, GCV treatment greatly increased the proportion of cells undergoing more than one round of replication (Figure 4h). Taken together, these results demonstrate the splenic environment (through the SASP) partially impairs T cell proliferation.

### 2.4 IR impairs macrophages and dendritic cells in the spleen

**Figure 5:**
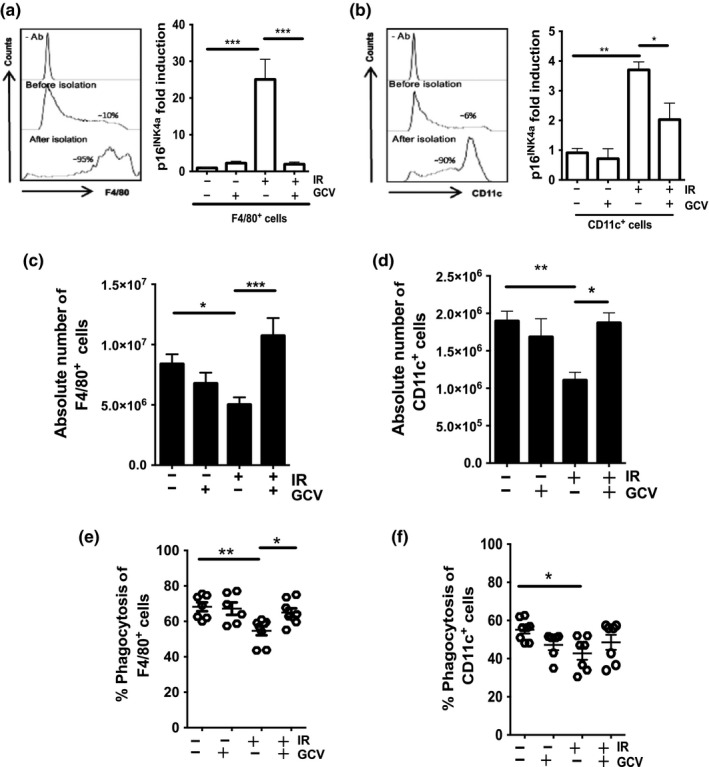
Impaired macrophage and DC counts and function in the splenic environment. (a, b) Shown are the purity (left panels) and quantification of *p16^INK4a^* mRNA levels (right panels) of isolated F4/80^+^ macrophages and CD11c^+^ DC cell populations as determined by flow cytometry and qPCR, respectively. 18S ribosomal RNA used as an internal control. (c, d) Shown is the quantification by flow cytometry of the absolute cell counts per spleens for F4/80^+^ and CD11c^+^ cell populations, respectively, collected from mice treated as indicated. Cell counts were determined 1 day following the last injection of GCV. (e, f) Quantification of the proportion of purified F4/80^+^ macrophages and CD11c^+^ DC populations capable of phagocytosis. Shown is the average ± *SEM* from *n* = 7–15 mice per group except for panels e and f where each dot represents counts from an individual mouse. The *ρ* value was determined by a one‐way ANOVA. ***p < 0.001; **p < 0.01; *p < 0.05

Macrophages and dendritic cells (DC) play a pivotal role in innate and adaptive immunity through their capacity to phagocyte, process, and present antigens. These cells exhibit more radio‐resistance than lymphocytes 1 week following exposure to IR (Figure S2 and S3). However, 8–9 weeks after IR, these cells (herein defined as F4/80^+^ for macrophages and CD11c^+^ for DC) display elevated levels of p16^INK4a^ expression and a decrease in absolute cell number compared to cells collected from nonirradiated animals (Figure 5a–d). In particular, macrophages display approximately 20‐fold increase in p16^INK4a^ expression which GVC injection completely abrogates. In contrast, DC only exhibit a threefold to fourfold increase in p16^INK4a^ expression which GVC only partially rescues. GVC administration restored the absolute cell count of both populations, with macrophage counts slightly higher (Figure 5c,d). However, phagocytes from irradiated mice exhibited reduced function as evidenced by their diminished capacity to take up a fluorescent substrate. Treatment of mice with GVC partially rescued macrophage capacity but not DC (Figure 5e,f). We suspect that this defect is intrinsic to irradiated cells, as we used magnetically purified populations of macrophages and DC in this assay (purity >90%). Moreover, we did not detect a reduction in the number of particles taken up by each cell, as determined by mean fluorescence (data not shown). We also observe a reduction in the expression of costimulatory molecules (CD80 and CD86) expressed on irradiated macrophages and DC compared to nonirradiated controls (Figure S4). Further studies are needed to evaluate whether such reduction is sufficient to drive impaired T cell proliferation. This result suggests that a sub‐population of cells lost their ability to phagocyte in contrast to a majority of cells with a slightly diminished capability.

## 3 DISCUSSION

Identification of the mechanisms involved in the loss of immune cell fitness, either during normal aging or following exposure to chemo/radiotherapy, is necessary for the development of a pharmacological treatment. Overall, we notice that exposure of mice to IR leads to a decrease in the counts of macrophage, dendritic and lymphoid cell populations in the spleen. Macrophages and DC represent a small proportion of myeloid cells, and these results are consistent with myeloid skewing we and others observed in the peripheral blood of irradiated mice (Carbonneau et al., 2012; Chang et al., 2016). Here, we provide evidence p16^INK4a^ expression increases the presence of a SASP in the irradiated spleen. We show previously (8–9 weeks) irradiated mice exhibit compromise proliferation of splenic T cells in vitro (upon allogenic stimulation) or in vivo (in response to an acute LCMV‐Arm infection). Defect in T cell proliferation appeared nonautonomous as CD3^+^ purified cells from irradiated spleens proliferate as expected in a MLR assay. These results suggest that increase in p16^INK4a^ expression (fourfold) is not sufficient to limit proliferation. Alternatively, perhaps only a small fraction of cells express higher level of p16^INK4a^ (Liu et al., 2019). Unfortunately, the limitation of the 3MR reporter genes (intensity of the luminescence/fluorescence signal) prevents the quantification of p16^INK4a^ expression at the single‐cell level. Despite relative purity (over 90%) of the CD3^+^ cell populations used in our studies, we cannot rule out the possibility that the increase originates from a few contaminating cells expressing high levels of p16^INK4a^ (such as macrophages).

We also observed that the irradiated splenic environment limits the proliferation of T cells in conditions with irradiated stimulator cells (CD‐1 mouse splenocytes). This observation stems with freshly irradiated CD‐1 compared to splenocytes isolated from p16‐3MR mice irradiated for as long as 8–9 weeks. We speculate freshly irradiated CD‐1 cells require time postirradiation to develop a SASP and an increase p16^INK4a^ expression, the latter delayed several weeks post exposure to IR (Le et al., 2010).

Our results also show that the SASP mediates part of the inhibitory effect induced by the irradiated splenic environment. We identified several SASP factors, but unable to distinguish whether a specific cytokine promoting the proliferation of T cells decreased or the secretion of an inhibitory molecule increased. A possible candidate‐IL‐10, an immunosuppressive cytokine, whose expression increased twofold in irradiated spleens and reduced following administration of GCV. Intriguingly, we observed the inhibitory impact of irradiated splenic environment on proliferation of purified CD3^+^ T cells more pronounced (55%) than on nonpurified splenic T cells (40%, see Figures 4b vs. 3b, respectively). This result likely due to addition of a small volume of purified CD3^+^ T cells in the MLR mix only marginally dilutes the irradiated environment.

Our MLR data performed in the absence of cellular interactions between effector cells and the irradiated splenocyte environment showed a smaller decrease in T cell proliferation compared to irradiated splenocytes in contact with effector cells (Figure 4e vs. b). A possible explanation for this difference we observed reduced phagocytosis capacity in macrophages and DC, a defect only restored in macrophages following elimination of p16^INK4a^‐positive cells. Whether a decrease in phagocytosis is sufficient to negatively impact T cell proliferation in the context of a MLR requires more study. We also observe a slight increase in the number of macrophages following the injection of GCV in irradiated mice, an unexpected result given the high level of p16^INK4a^ expression in this population. We speculate the increase may result from peripheral macrophages recruited to the spleen in response to GCV‐induced cell death. Recent data demonstrate immune stimuli modulate the expression of p16^INK4a^ in macrophages, suggesting a complex regulation in these cells and more work required to delineate complex regulation (Hall et al., 2017).

Studies in aging mice and humans show an increase in the proportion of regulatory T cells (T regs) that impair immune function (Sharma, Dominguez, & Lustgarten, 2006; Simone, Zicca, & Saverino, 2008). We quantified the proportion of T regs in the spleen and found their number sharply decreased in irradiated mice (with and without treatment with GCV), suggesting these cells are unlikely to negatively impact proliferation of T cells (Figure S5). Finally, IR may act on the stromal architecture of the spleen stroma. Indeed, we observe a significant increase in p16^INK4a^ expression and decrease in absolute cell counts following the injection of GCV in sub‐populations (gp38^+^ and CD35^+^) of splenic stromal cells (Figure S6). Hence, while we show that the SASP negatively affects proliferation of splenic T cells, the overall impact of IR on the spleen function may alter multiple signaling pathways and is likely multifactorial.

In conclusion, we demonstrate that elimination of p16^INK4a^ expressing cells within the splenic environment improves some immune cell functions. Studies to evaluate whether newly developed senolytic drugs (Baar et al., 2017) also increase the fitness of immune cells in aged mice or mice receiving radiotherapy and/or chemotherapy may provide additional information.

## 4 MATERIALS AND METHODS

### 4.1 Animals and treatments

*p16*‐3MR mice were kindly donated by Dr. Judith Campisi (Buck Institute) and breed on site according to a material transfer agreement (Demaria et al., 2014). All in vivo manipulations were approved by the Comité Institutionnel des Bonnes Pratiques Animales en Recherche of the CHU Ste‐Justine. 12‐ to 14‐week‐old p16^INK4a^‐3MR mice were exposed to X‐rays at the single sublethal dose of 6.5 Gy (1 Gy/min) using a Faxitron CP‐160. GCV was administrated daily by intraperitoneal (*i.p*.) injections for 5 consecutive days at a dose of 25 mg/kg in 1X‐PBS (Sigma).

### 4.2 Viral infection

Mice were injected *i.p.* with 2 × 10^5^ pfu of lymphocytic choriomeningitis virus (LCMV) strain Armstrong (LCMV‐Arm) to generate acute infection. Seven days postinfection, spleens were harvested from infected mice and filtered through a 70 μm pore‐size cell strainer (Falcon, Franklin Lakes, NJ) and centrifuged at 200 *g* for 5 min at 4°C. Splenocytes were treated with NH_4_Cl to remove erythrocytes. For all experiments, dead cells were stained with fixable LIVE/DEAD Aqua (Catalog, L3496, Life Technologies) and excluded from the analysis. For granzyme B release, splenocytes were restimulated in vitro for 4 hr with a cognate gp33 peptide (0.1 mM) in the presence of GolgiStop (Catalog, 554724, BD). Cells were then fixed and permeabilized using the Cytofix/Cytoperm kit (Catalog, 554722, BD) and stained for granzyme B (Clone GRB05, Life Technologies). For nuclear staining, splenocytes were processed directly ex vivo. Cells were Fc‐blocked, and extracellular staining was performed in 50–100 μl of PBS with 2% (vol/vol) FBS for 20 min on ice before fixation. Cells were fixed with Cytofix/Cytoperm (Catalog, 554722, BD) followed by intracellular Ki67 staining (Clone SolA15, Bioscience).

### 4.3 Bioluminescence

To detect luminescence from the 3MR gene cassette, mice were anesthetized using isoflurane and injected *i.p.* with water‐soluble coelenterazine (CTZ; Catalog, 3031, NanoLight Technology™) at a concentration of 1 mg/ml in 1X‐PBS. Mice were imaged using the Epi‐Fluorescence & Trans‐Fluorescence Imaging System (Labeo Technologies) 14 min postinjection. Mice were euthanized, spleens surgically removed, and bioluminescence levels measured ex vivo in a solution of 1 mg/ml of CTZ.

### 4.4 Gene expression

RNA was extracted from spleens and from isolated CD3^+^, B220^+^, gp38^+^, CD35^+^, CD11c^+^, and F4/80^+^ cell populations using the RNeasy^®^ Mini or Micro Kit (Qiagen). Cells were purified using EasySep™ PE Positive Selection Kit (Catalog, 18551, StemCell Technologies) according to the manufacturer's instructions. RNA was reverse‐transcribed using the QuantiTect Reverse Transcription Kit. Quantitative differences in gene expression were determined by real‐time quantitative PCR using SensiMixTM SYBR Low‐ROX (Quantace) and the MxPro QPCR software (Stratagene). Values are presented as the ratio of target mRNA to 18S rRNA, obtained using the relative standard curve method of calculation.

### 4.5 Flow cytometric analysis

To obtain absolute cell counts from various populations, spleens were processed in 1X‐PBS containing 2% FBS and mechanically disrupted with flat portion of a plunger from a 5 mL syringe. Samples were incubated with collagenase D for 30 min (Catalog, 11088866001, Roche). Splenic cell suspension was passed through a 70 μm pore‐size cell strainer (Falcon, Franklin Lakes, NJ) and centrifuged at 200 *g* for 5 min at 4°C. Splenic cell counts were determined using Count Bright^®^ Absolute Counting Beads (Catalog, C36950, Thermo Fisher) and analyzed using the Becton Dickinson Immunocytometry Systems (BD LSR‐Fortessa™). Briefly, red blood cells were lysed by adding 5 ml of lyse solution (0.14 M NH4CL, 0.02 M Tris‐HCl, pH 7.2). The tubes were incubated at room temperature (RT) for 5 min and washed twice with 10 ml of Roswell Park Memorial Institute (RPMI) medium containing 10% fetal bovine serum (FBS). Cells were centrifuged and pellet re‐suspended in 3 ml of 1X‐PBS from which 10 µl of cell suspension was stained with fluorophore‐conjugated antibodies all purchased from BioLegend: F4/80 (clone BM8), CD3 (clone 17A2), CD4 (clone GK1.5), CD8α (clone 53–6.7), CD11b (clone M1/70), CD11c (clone N418), CD35 (clone 7E9), gp38 (clone 8.1.1), CD31 (clone 390), PDGFR (clone APA5), and CD45 (clone 30‐F11).

### 4.6 In vitro phagocytosis assay

Splenic CD11c^+^ DCs and F4/80^+^ macrophages were purified using the EasySep™ PE Positive Selection Kit according to the manufacturer's instructions. Purified cells (purity of ~90%) were used at a concentration of ~1 × 10^5^ cells/ml in RPMI supplemented with 10% FBS and 1% antibiotics containing Phrodo™ Green Zymosan A particles (Catalog, P35365, Thermo Fisher) for 90 min at 37C in a 5% CO_2_ incubator. Phagocytosis of Zymosan A particle by purified CD11c^+^ DCs and F4/80^+^ macrophages was determined by flow cytometry on a minimum of 50,000 events.

### 4.7 Multiplex cytokine analysis

The splenic cell secretome was quantified by Eve Technologies (Calgary, Canada) using the 31‐Plex Mouse Cytokine Array/Chemokine Array. Splenocyte samples were processed according to Eve Technologie's recommendations. Briefly, 1 × 10^7^ splenocytes were pelleted and washed twice with 1X‐PBS; then, cells were lysed with radioimmunoprecipitation assay (RIPA) buffer on ice for 10 min (20 mM Tris‐HCl, pH 7.5, 0.5% Tween‐20, 150 mM NaCl) with 1% protease inhibitors (PI). Lysates were centrifuged at 10,000 *g* for 10 min at 4°C and supernatants transferred to a new tube and normalized with 1X‐PBS to 0.5 mg/ml of proteins.

### 4.8 T cell proliferation assays

T cell proliferation was evaluated by an allogenic mixed lymphocyte reaction (MLR) assay. Briefly, cells were harvested from spleens of p16‐3MR mice irradiated 8–9 weeks earlier and untreated or treated with GCV for 5 consecutive days prior to sacrifice. Splenocytes or purified CD3^+^ cells were labeled with CellTrace™ 6‐carboxy‐succinimidyl‐fluorescein‐ester dye (CFSE) and used as responder. CD3^+^ cells were isolated by negative selection using the EasySep Mouse T Cell Isolation Kit (Catalog, 19851, STEMCELL). The purity of CD3^+^ cells was determined by flow cytometry using a PE‐conjugated anti‐CD3 antibody (Catalog, 100240, BioLegend). Splenocytes obtained from the outbred CD‐1^®^IGS mouse strain (Charles River) were used as stimulator and irradiated at a dose of 30 Gy. Mixed lymphocyte reactions were set up with 1 × 10^5^ CFSE‐labeled p16‐3MR responder cells and 2 × 10^5^ freshly irradiated allogenic CD‐1 stimulator splenocytes in round‐bottom 96‐well plates, at 37°C, 5% CO_2_ for 3 days. Alternatively, responder and stimulator cells were separated by a 8.0 µm Transwell^®^ system (Catalog, 3422, Costar). Where indicated, responders and stimulators were mixed with a splenic “environment” consisting of freshly isolated splenocytes depleted of CD3^+^ cells using a magnetic‐bead‐mediated positive PE selection kit (Catalog, 19851, STEMCELL). Depletion efficiency measured by flow cytometry was over 90%. Negatively isolated p16‐3MR CFSE‐labeled CD3^+^ cells were incubated in a CD3^+^‐depleted environment at a proportion of 1:5. The combination of CFSE‐labeled CD3^+^ cells and CD3^+^‐depleted splenocytes was incubated with allogenic CD‐1 splenocytes at a proportion of 1:2. Where indicated, the proliferation of p16‐3MR CFSE‐labeled CD3^+^ responder cells was also induced using the Dynabeads^®^ mouse T‐activator CD3/CD28 system (11456D, Fisher).

### 4.9 Statistical analysis

GraphPad Prism 7 software was used for statistical analysis; ρ values on multiple comparisons were calculated using one‐way analysis of variance (ANOVA) with Bonferroni post hoc test.

## CONFLICT OF INTEREST

None declared.

## AUTHOR CONTRIBUTIONS

L.P., A.E., N.V., M‐L.G., D.M., A.D., S.B., G.M.B., and O.L. performed experiments. L.P., S.B., H.D., and C.B. designed the studies. M.D. provided reagents and expertise. L.P. and C.B. wrote the manuscript. A.D. edited the manuscript.
