# Supplementary material for: Restored immune cell functions upon clearance of senescence in the irradiated splenic environment
Source: Aging Cell. 2019 May 31;18(4):e12971. doi: 10.1111/acel.12971 (PMC6612633; doi:10.1111/acel.12971)
Supplement: Supplementary file 1 [file ACEL-18-e12971-s001.pdf]

Figure S1. Palacio *et al*.

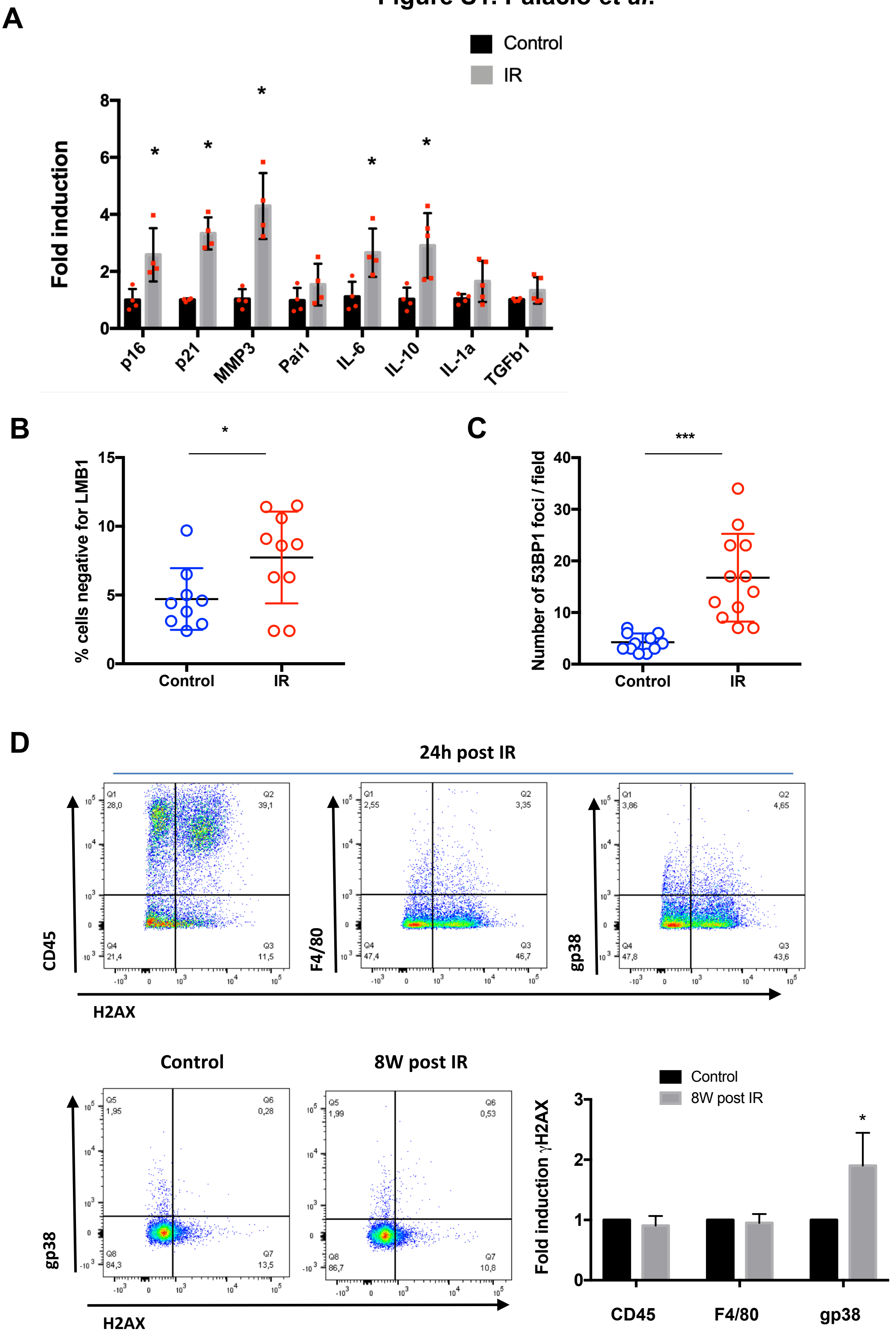

Supplementary figure 1. Expression of senescence markers in the irradiated spleen. (**a**) Mice were irradiated (6.5 Gy) and 8 weeks later spleens were collected and RNA purified from total cells. Quantification of *SASP factors* was determined by qPCR. Beta-2 microglobulin was used as an internal control. Shown is the mean +/- SEM of n=4 mice per group. (**b)** Loss of lamin B1 expression was determined by staining dissociated splenic macrophages. Of note, no difference in lamin B1 expression was noticed in T and B cells (not shown). Each dot represents the proportion of cells per field with no lamin B1 expression. A minimum of 100 cells/field with 3-4 fields/mouse for n=3 mice were used. (**c)** Increased 53BP1 DNA damage foci in spleen sections collected from irradiated mice. Each dot represents the average number of cells per field collected from 3 individual mice (minimum 100 cells/field with 3-4 fields/mouse). (**d**) Detection by flow cytometry of the proportion of splenic cells positive for γH2AX. Spleens were dissociated at the single cell level and gated on different sub-populations (CD45+, F4/80+ and gp38+). Top row shows the staining of all cell types 24 hours after IR. Only the gp38 stromal population had a residual increase in γH2AX 8 weeks post IR. Shown is the mean +/- SEM of n=3 mice per group. *p<0.05, ***p<0.001

Figure S2. Palacio *et al*.

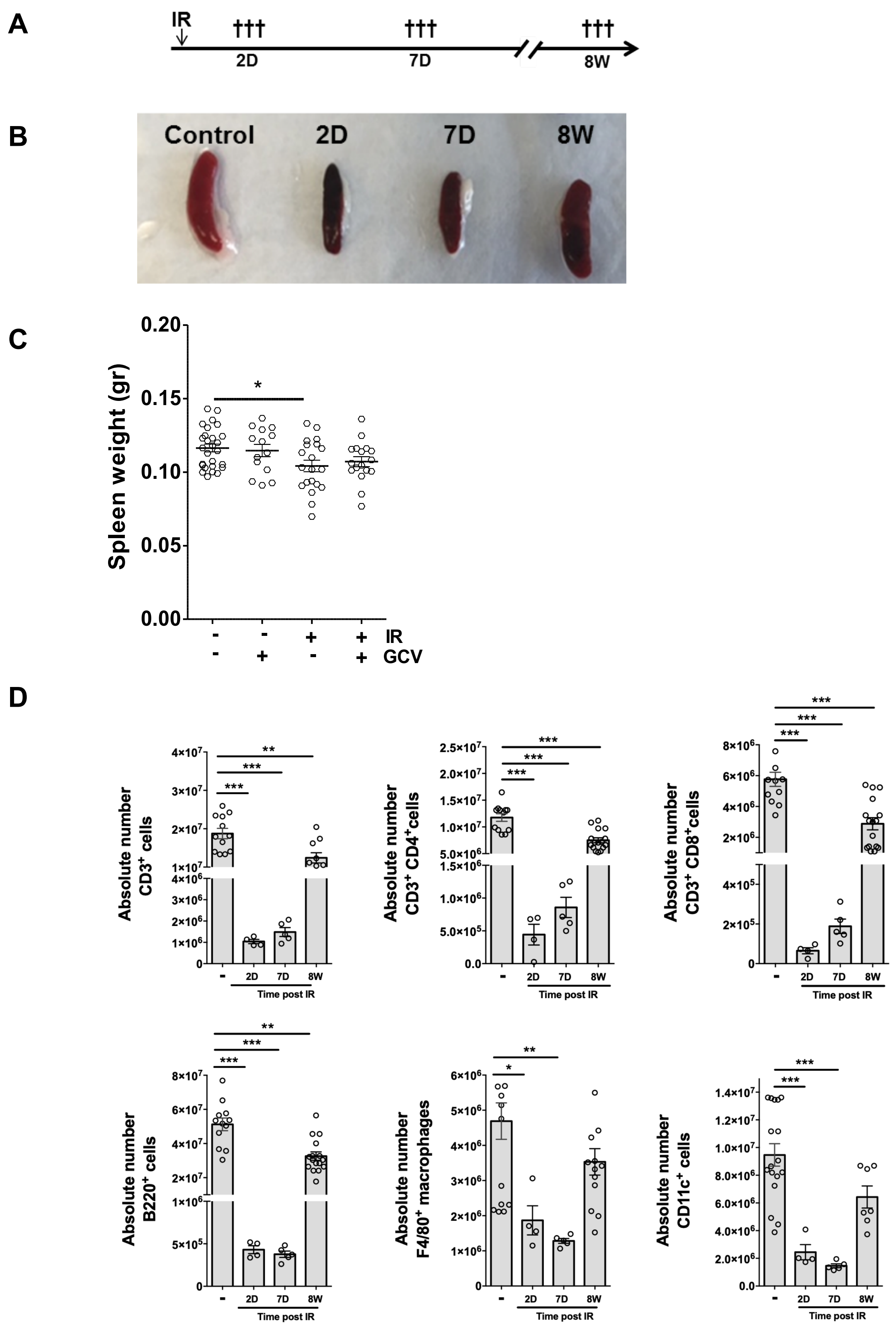

Supplementary figure 2. Splenic cell counts following exposure to IR. (**a**) Schematic of the experimental design. 12 week-old p16-3MR mice were exposed to 6.5 Gy total body irradiation and were sacrificed two days, seven days or 8 weeks later. (**b)** Representative photographs of freshly excised spleens from control and irradiated mice at the indicated time post exposure. (**c**) Spleen weight expressed in grams (gr) from p16-3MR mice exposed (+) or not (-) to IR and/or GCV. (**d)** Quantification by flow cytometry of the absolute cell counts for $CD3^+$, $CD3^+CD4^+$, $CD3^+CD8^+$, $B220^+$, $F4/80^+$ and $CD11c^+$ populations per spleen of controls and irradiated mice. Shown is the average +/- SEM from n= 4-15 mice per group were each dot represents the weight of an individual mouse. The *ρ* value was determined by a One-way ANOVA. *** $p<0.001$; **$p<0.01$; *$p<0.05$.

Figure S3. Palacio *et al*.

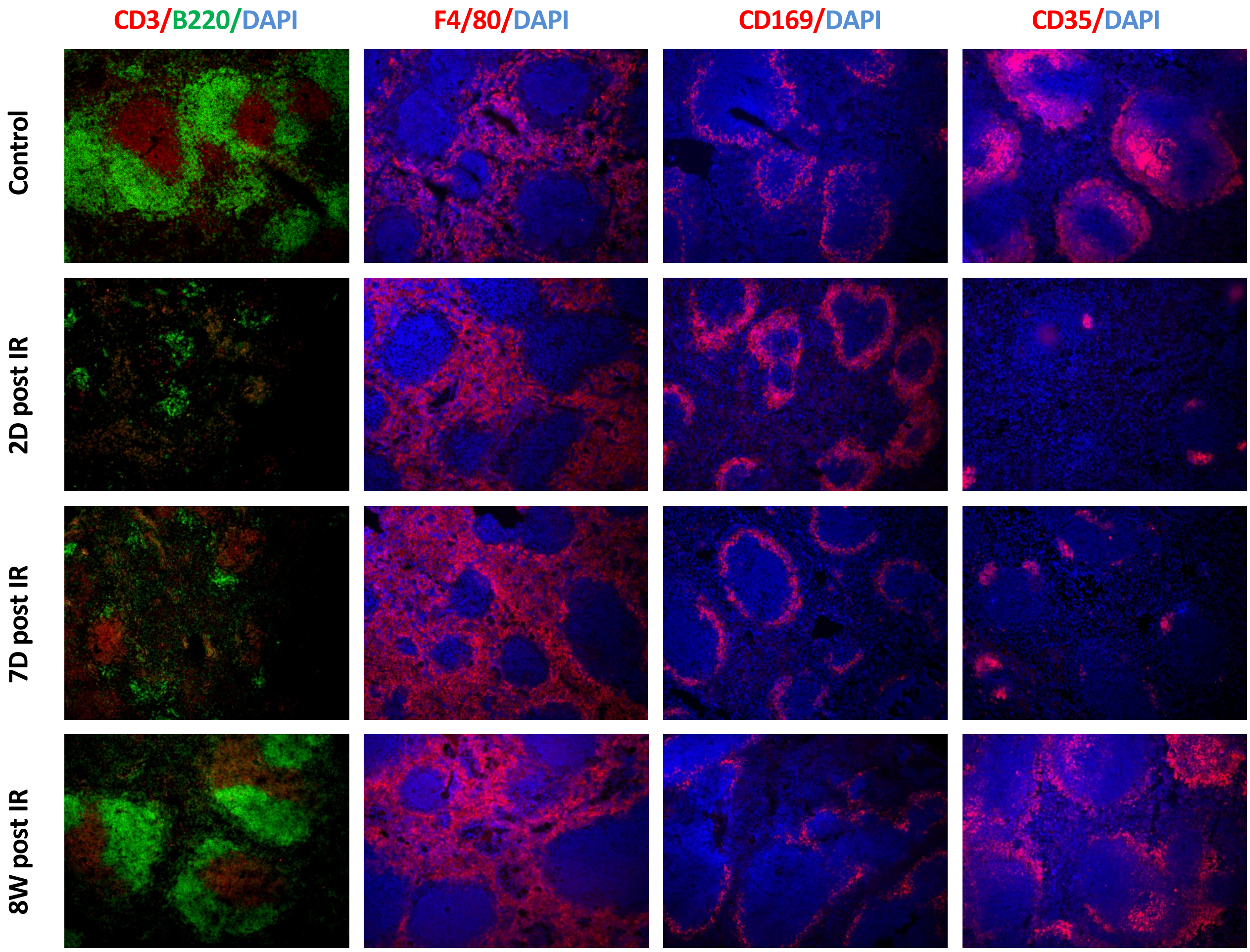

Supplementary Figure 3. Short and long-term Impact of IR on splenic cell sub populations. Representative images of T cells (CD3 in red), B cells (B220 in green), macrophages (F4/80 in red), macrophages from the marginal zone (CD169 in red) and stromal cells from the white pulp ($CD35^{+}$ in red) on spleen sections at the indicated time after exposure to IR (6.5 Gy). Shown in blue is DAPI. d = days, w = week.

Figure S4. Palacio *et al*.

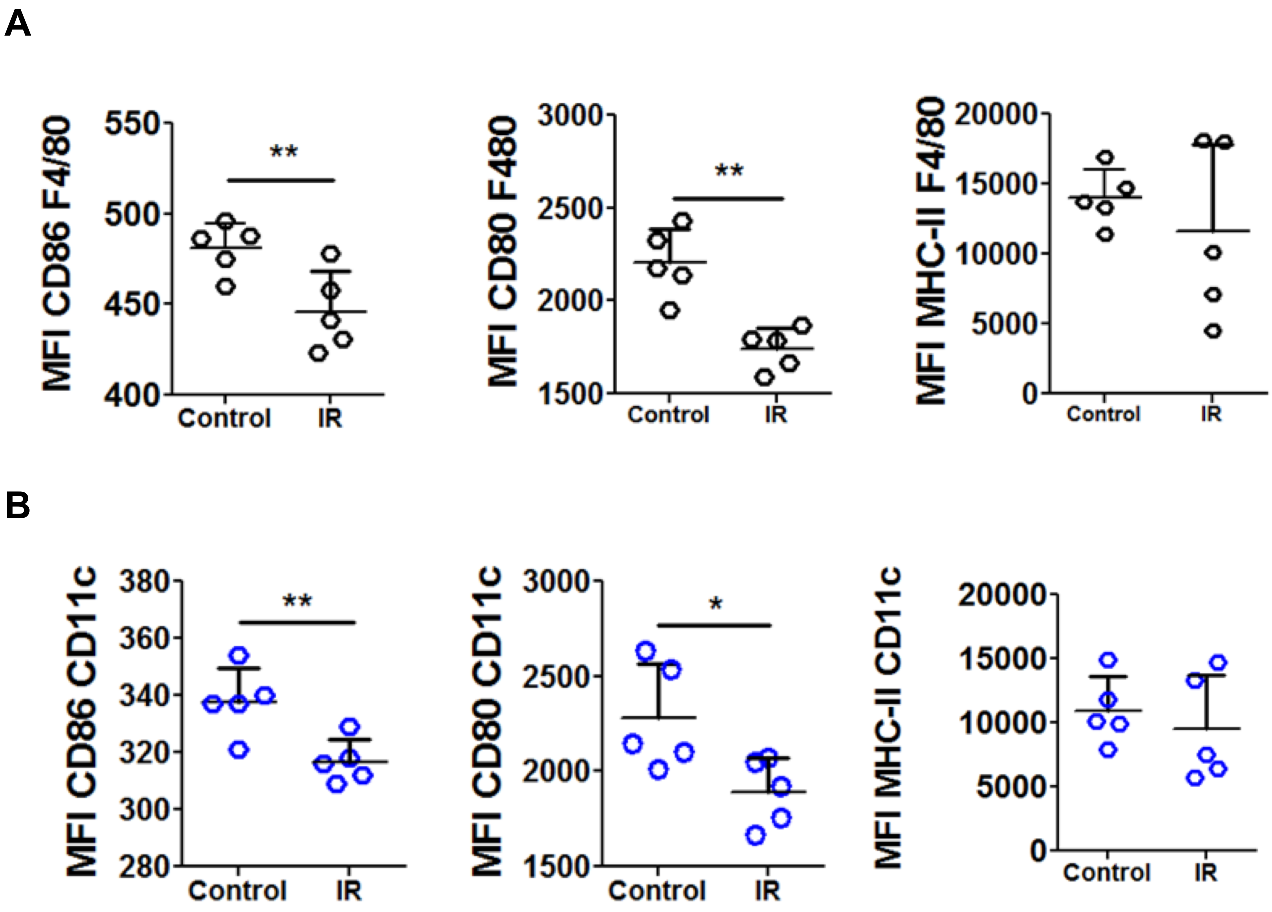

Supplementary Figure 4. Exposure to IR compromises the expression of costimulatory molecules on APC. Quantification of the expression levels of CD86 and CD80 costimulatory molecules and MHC-II at the surface of on F4/80^{+} macrophages (**a**) and CD11c^{+} DC (**b**) populations. Expression levels were determined by flow cytometry using the mean fluorescence intensity (MFI). The data are presented as mean +/- SEM; n= 5 mice per group with each dot representing measures from an individual mouse ***p*<0.01; **p*<0.05.

Figure S5. Palacio *et al*.

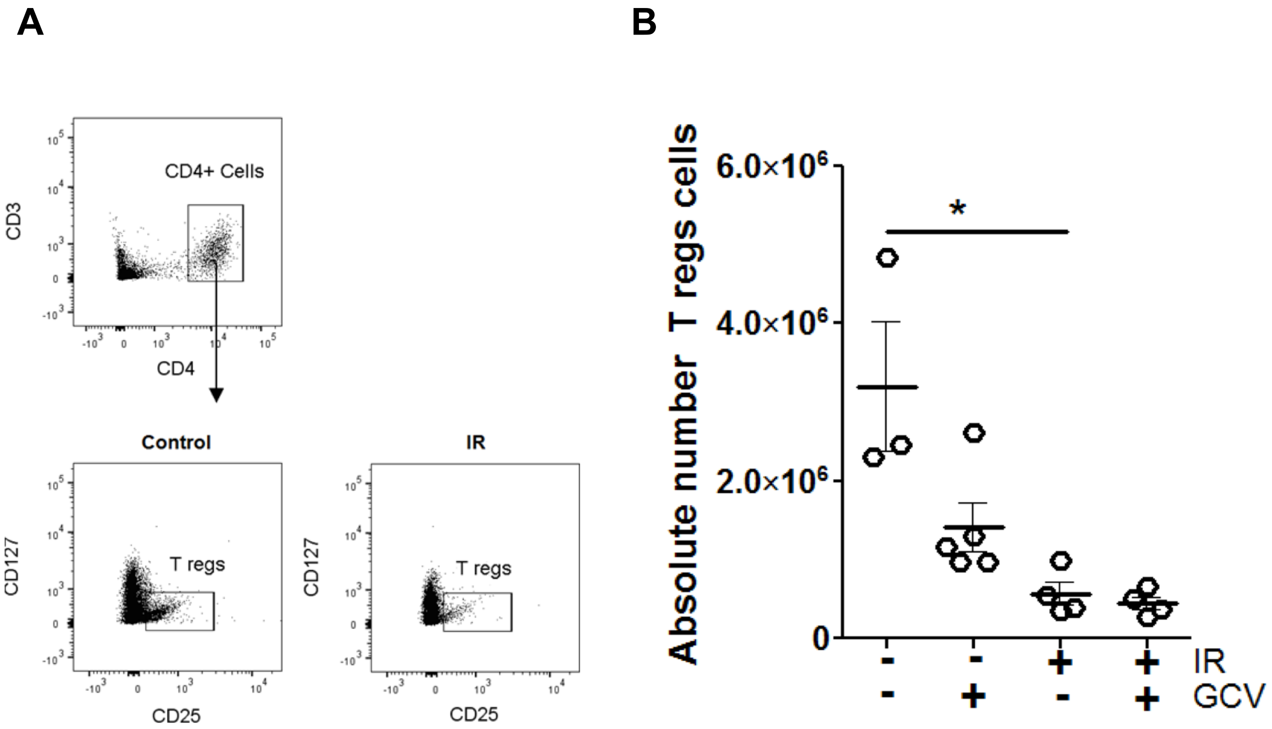

Supplementary Figure 5. Decreased number of T regulatory cells in the irradiated spleen of p16-3MR mice is not restored by GCV. Quantification of the absolute count of T regs in the spleen of irradiated p16-3MR mice treated or not with GCV for 5 days. **(a)** T regs counts were determined by flow cytometry using beads on CD3^{+}, CD127^{-} and CD25^{+} gated cells. **(b)** Absolute counts of T reg cells per spleen as determined by flow cytometry. n=3-5 mice per group with each dot representing counts from an individual mouse. *p<0.05.

Figure S6. Palacio *et al*.

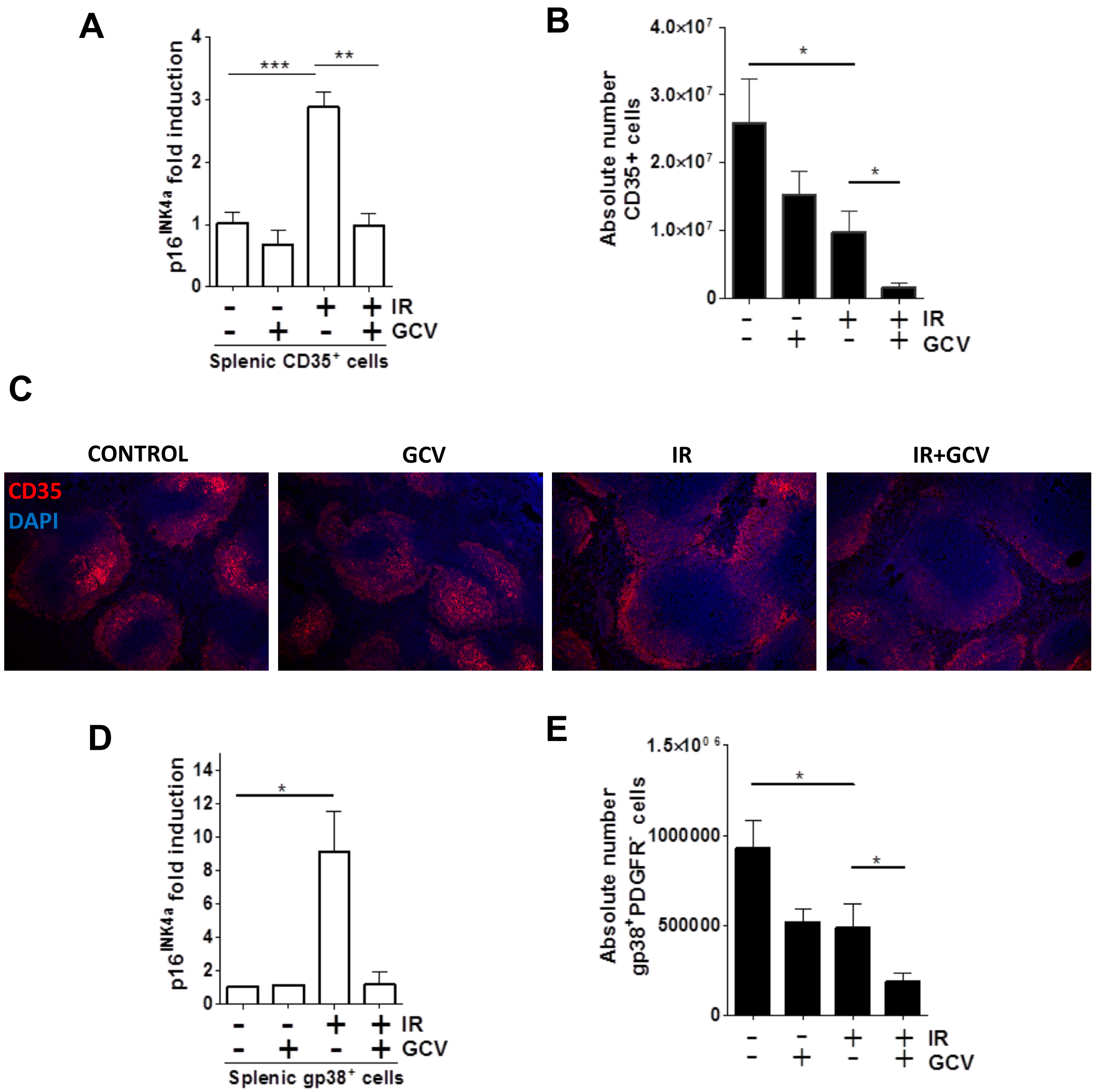

Supplementary Figure 6. Exposure to IR impacts splenic stromal cell sub-populations. Quantification of *p16^{INK4a}* mRNA levels (**a**) and absolute cell counts per spleen **(b)** of CD35^{+} isolated stromal cells as determined by qPCR and flow cytometry respectively. 18S ribosomal RNA was used as an internal control. Cells populations were purified by magnetic cell sorting and purification was over 90% (data not shown). **(c)** Representative images of CD35^{+} staining (in red – clone 7E9 from Biologend) on spleen sections from mice treated as indicated. Shown in blue is DAPI. Quantification of *p16^{INK4a}* mRNA levels (**d**) and absolute cell counts per spleen **(e)** of gp38^{+} PDGFR^{-} isolated cells is shown. The data are presented as mean +/- SEM of n=6 mice per group. The *p* value was determined by a One-way ANOVA. *p*<0.001; ***p*<0.01; **p*<0.05.
